# Low Temperature Synthesis of Lithium-Doped Nanocrystalline Diamond Films with Enhanced Field Electron Emission Properties

**DOI:** 10.3390/nano8090653

**Published:** 2018-08-24

**Authors:** Kamatchi Jothiramalingam Sankaran, Kalpataru Panda, Ping-Yen Hsieh, Paulius Pobedinskas, Jeong Young Park, Marlies K Van Bael, Nyan-Hwa Tai, I-Nan Lin, Ken Haenen

**Affiliations:** 1 Institute for Materials Research (IMO), Hasselt University, 3590 Diepenbeek, Belgium; paulius.pobedinskas@uhasselt.be (P.P.); marlies.vanbael@uhasselt.be (M.K.V.B.); 2 IMOMEC, IMEC vzw, 3590 Diepenbeek, Belgium; 3 Center for Nanomaterials and Chemical Reactions, Institute for Basic Science (IBS), Daejeon 34141, Korea; phy.kalpa@gmail.com (K.P.); jeongypark@kaist.ac.kr (J.Y.P.); 4 Department of Materials Science and Engineering, National Tsing Hua University, Hsinchu 30013, Taiwan, China; qqwweerreewwqq@hotmail.com (P.-Y.H.); nhtai@mx.nthu.edu.tw (N.-H.T.); 5 Graduate School of EEWS, Korea Advanced Institute of Science and Technology (KAIST), Daejeon 34141, Korea; 6 Department of Physics, Tamkang University, Tamsui 251, Taiwan, China; inanlin@mail.tku.edu.tw

**Keywords:** nanocrystalline diamond, lithium, low temperature, field electron emission

## Abstract

Low temperature (350 °C) grown conductive nanocrystalline diamond (NCD) films were realized by lithium diffusion from Cr-coated lithium niobate substrates (Cr/LNO). The NCD/Cr/LNO films showed a low resistivity of 0.01 Ω·cm and excellent field electron emission characteristics, viz. a low turn-on field of 2.3 V/µm, a high-current density of 11.0 mA/cm^2^ (at 4.9 V/m), a large field enhancement factor of 1670, and a life-time stability of 445 min (at 3.0 mA/cm^2^). The low temperature deposition process combined with the excellent electrical characteristics offers a new prospective for applications based on temperature sensitive materials.

## 1. Introduction

Nanocrystalline diamond (NCD) films grown by chemical vapor deposition, possessing unique and advantageous properties such as high hardness, high chemical inertness, and negative electron affinity (NEA), can be a good candidate for device applications [1,2]. Despite these marvelous properties, the absence of highly conducting NCD confines the potential for the use of this material in electron emission devices. Fortunately, doping NCD with *p*-type (e.g., boron) [3] or *n*-type (e.g., nitrogen, phosphorus etc.) [4,5] dopants can render the films conductive. Particularly, *n*-type doped NCD films reveal highly efficient field electron emission (FEE) characteristics compared to the *p*-type ones [3,6]. Nevertheless, a high-substrate growth temperature of above 800 °C is required to stimulate the *n*-type doping process, especially when nitrogen and phosphorus are used as dopants [4,5,6], which is not well-suited for the device fabrication processes. Fabrication of highly conducting *n*-type NCD films at low temperature are desired for the development of diamond-based electronics.

Lithium (Li) is a possible shallow *n*-type dopant, as Li is anticipated to occupy interstitial sites in the diamond lattice [7]. Nevertheless, the doping of Li into diamond via ion implantation [8], adsorption [9], diffusion [10,11], or addition of Li to the gas phase during growth [12,13] were not effective for the fabrication of a highly conducting diamond films, and hence the FEE results obtained so far from these films were also not satisfactory [14]. Freestanding ultrananocrystalline diamond (UNCD) films grown directly on lithium niobate (LNO) substrate have been an exception [15]. However, the highly conducting UNCD films thus obtained were very fragile and detached from the substrate easily, avoiding their use for the fabrication of electronic devices.

In this context, a thin chromium (Cr) interlayer was used for maintaining the integrity of NCD films grown on LNO substrates, which served as a possible source of Li doping. Highly-conducting Li-doped NCD films were synthesized at a very low temperature of about 350 °C, and an enhanced FEE behavior and plasma illumination (PI) properties for NCD films were demonstrated. The potential mechanism for the enhanced FEE and PI properties of Li-doped NCD films was discussed based on the investigation using Raman spectroscopy, X-ray photoelectron spectroscopy (XPS), and secondary ion mass spectroscopy (SIMS).

## 2. Materials and Methods

The Y-cut oriented LNO single crystal substrates (1 × 1 cm^2^ in sized; Crystal GmbH, Berlin, Germany) polished to a smoothness of 0.9 nm were used for growing NCD films. A 50 nm thick Cr layer was deposited on the LNO substrates to improve the adhesion between the LNO substrates and the NCD films. The Cr-coating was deposited by a home-built DC-pulsed sputtering system using a radio frequency power of 200 W in Ar gas for 10 min. The Cr-coated LNO (Cr/LNO) substrates were then nucleated with a water-based state-of-the-art colloidal suspension of ultradispersed detonation diamond (Nano-Carbon Institute Co., Ltd., Nagano, Japan; zeta potential of (45 ± 5) mV and particle size of 6–7 nm) via drop casting and subsequent spin-drying [16]. The growth of ~90 nm thick NCD layers, designated as NCD/Cr/LNO films, was performed in a linear antenna microwave plasma enhanced chemical vapor deposition (LA CVD) system using plasma containing 10% methane in hydrogen. The film thickness was monitored by in situ laser reflection interferometry. The microwave power and pressure were 3000 W and 0.21 Torr, respectively. The substrates were heated up due to the bombardment of the plasma species and the substrate temperature was measured by a single color optical pyrometer with an optical emission coefficient of 0.3 during NCD film deposition. The measured substrate temperature was about 350 °C, which is even lower than the temperature used for the growth of freestanding UNCD films on LNO substrates [15] and is compatible with a Si-device fabrication process. The key necessity for diamond growth at low temperature is a high-plasma density at a low-gas pressure, which leads to a low thermal loading of the substrate materials. While these conditions are not within reach for resonance cavity plasma systems, the LA CVD system enables the deposition of high-quality diamond films on larger areas with satisfactory growth rates, while allowing an easy up-scaling and industrial implementation [17]. To facilitate the comparison, NCD films were also grown on Si-substrates covered with a similar Cr-coating, using the same deposition parameters. The obtained diamond films were designated as NCD/Cr/Si films.

The surface morphology and the crystalline quality of the NCD films were investigated by scanning electron microscopy (SEM; FEI Quanta 200 FEG microscope from ThermoFisher Scientific, Hillsboro, OR, USA), atomic force microscopy (AFM; multi-mode VIII with a Nanoscope V controller from Bruker, Billerica, MA, USA) and micro-Raman spectrum (Horiba Jobin-Yvon T64000 spectrometer, Paris, France; λ = 488.0 nm). The surface chemical bonding characteristics of the films were investigated by XPS (PHI 1600, Physical Electronics, Chanhassen, MN, USA). Al Kα radiation with energy of 1486.74 eV and an energy resolution of 0.47 eV was used as a probe for evaluating the chemical bonding fractions of different phases on the NCD film surfaces. An elemental depth profile analysis of the NCD/Cr/LNO films was carried out using SIMS (TOF-SIMS5, ION-TOF GmbH, bismuth ion source). The resistivity of the NCD films was measured using four-point probe measurements. Room temperature FEE properties of NCD films were measured using a tunable parallel-plate setup, in which the anode is a Mo-rod of 3 mm in diameter and the cathode-to-anode distance “*d*” was controlled to be around 66 µm by a micrometer. The current-voltage characteristics were acquired at a pressure below 10^−6^ Torr using a Keithley 237 electrometer.

## 3. Results and Discussion

The featured NCD/Cr/LNO and NCD/Cr/Si films were subjected to four-point probe measurements with the measuring probes directly in contact with the surface of the films to measure the electrical resistivity. It was found that the resistivity (ρ) values of NCD/Cr/LNO and NCD/Cr/Si films were 1 × 10^−2^ and 4.5 × 10^3^ Ω·cm, respectively, viz. a high conductivity of NCD films was obtained through the use of Cr/LNO substrates. Interestingly, the NCD/Cr/LNO films deposited at 350 °C exhibited low resistivity comparable to that of the freestanding UNCD films grown on LNO substrates (ρ = 1.2 Ω·cm) [15].

**Figure 1:**
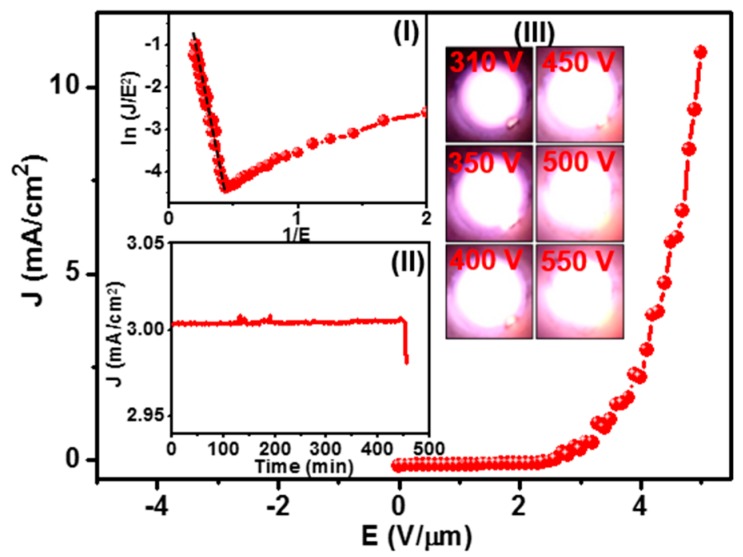
Field electron emission properties (current density-applied field, *J*-*E*, curve). Inset I shows the corresponding Fowler–Nordheim (F–N) plot, inset II shows the life-time stability measurement (*J*-time curve) for NCD films grown on Cr-coated LNO (NCD/Cr/LNO films, while inset III shows the plasma illumination (PI) images versus voltage applied to the microplasma devices using NCD/Cr/LNO films as cathode materials.

Figure 1 shows the FEE properties of the NCD/Cr/LNO films, whereas the inset I of Figure 1 indicates the corresponding Fowler–Nordheim (F–N) plot. The FEE parameters, including the turn-on field (*E*_0_) and FEE current density (*J*), were extracted from the *J*-*E* curves obtained at *d* = 66 μm (Figure 1), where *E* is the applied field, using the F-N equation [18]. The *E*_0_ was designated as the point of intersection of the straight lines extrapolated from the low-field and high-field segments of the F-N plots, viz. ln(*J*/*E*^2^) versus 1/*E* curves (inset I, Figure 1). The *E*_0_ value for inducing the FEE process is around 11.8 V/μm for NCD/Cr/Si films (curve II, Figure S1a of the supplementary information), whereas the *J* value is around 6.4 mA/cm^2^ at an applied field of 20.0 V/μm. In contrast, the NCD/Cr/LNO films required a much smaller field to turn on the FEE process, i.e., an *E*_0_ value of 2.3 V/μm and high *J* value of 11.0 mA/cm^2^ at an applied field of 4.9 V/μm (Figure 1). Moreover, the field enhancement factor (*β*) of the emission sites can be evaluated from the slope (*m*) of the F–N plot, viz. *m* = −*ϕ*^3/2^/*β* where *ϕ* is the work function of the diamond materials. The F-N plot in the inset I of Figure 1 shows that the *β* value of the NCD/Cr/LNO films is *β*_NCD/Cr/LNO_ = 1670, whereas the inset of Figure S1a in the supplementary information indicates that the *β*-value of NCD films is *β*_NCD/Cr/Si_ = 980, revealing a larger field enhancement factor value for the NCD/Cr/LNO films. Moreover, by tuning the cathode-anode separation distance, we characterized the FEE properties of the NCD/Cr/LNO films, in the range 66 μm < *d* < 120 μm. Figure S1b of the supporting information shows the *J*-*E* curves with corresponding F–N plots measured for different *d* values for NCD/Cr/LNO films. We notice that, as expected, by increasing the distance of the tip from the surface, higher applied field are necessary to extract electrons from NCD/Cr/LNO films. It is to be noted here that *E*_0_ and *β* values depend on the absolute cathode-anode separation [19,20].

Moreover, the life-time (τ) stability measurements of NCD/Cr/Si and NCD/Cr/LNO films were evaluated by measuring the *J* versus time curves of these films. Inset II of Figure 1 shows that, ignoring short-term fluctuations owing to adsorption and desorption of residual gas molecules and diffusion of adsorbed species on the emitter surface, the emission current density variations corresponding to *J* of 3.0 mA/cm^2^ were recorded over a period of 445 min for NCD/Cr/LNO films (at a working field of 4.0 V/μm), before the start of degradation. In contrast, the NCD/Cr/Si films show emission current variations recorded for a period of only 215 min under the same test current density of 3.0 mA/cm^2^ (at a working field of 18.9 V/μm) (curve II, Figure S1c of supplementary information). Consequently, the NCD/Cr/LNO films exhibit a far more superior FEE behavior, viz., lower *E*_0_, higher *J* and longer τ as compared with the other Li-doped diamond based field emitters reported in the literature, which are summarized in Table 1 [14,15]. Notably, in Table 1, the FEE properties of NCD/Si and NCD/Cr/Si films (shown as curves I and II, Figure S1a,c of Supplementary Materials) were included to facilitate the comparison.

**Figure 2:**
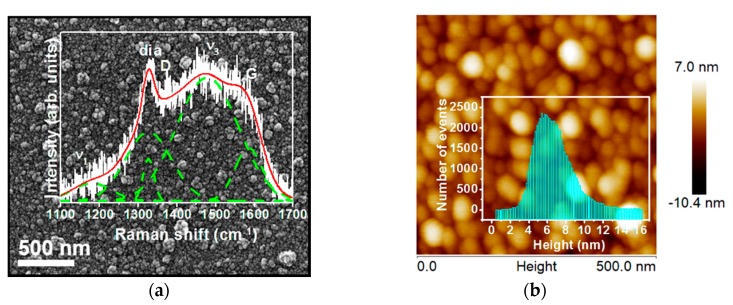
(**a**) SEM and (**b**) AFM micrographs of the NCD/Cr/LNO films, showing a small and uniform grain size distribution in the films. The inset in (**a**) shows the Raman spectrum (*λ* = 488.0 nm), whereas the inset in (**b**) shows a histogram for the grain size distribution of the films.

The SEM image shown in Figure 2a depicts that the NCD/Cr/LNO films have an equi-axed, nano-sized, granular structure. Moreover, Figure 2b shows an AFM image taken at ambient conditions along with a histogram (inset, Figure 2b) indicating that the average grain size of the NCD/Cr/LNO films is around 30 nm with a very narrow size distribution. The root mean square surface roughness values are ranged between 2–3 nm. The surface morphologies of the NCD/Si and NCD/Cr/Si films are like those of NCD/Cr/LNO films (Figure S2 in supplementary information). It is to be noted that the roughness values were obtained from the raw data without any smoothing or flattening processes.

The inset in Figure 2a shows the micro-Raman spectrum of the NCD/Cr/LNO films. Peaks at around 1190 cm^−1^ and 1470 cm^−1^ are ascribed to the υ_1_ and υ_3_-modes of *trans*-polyacetylene present at the grain boundaries [21]. A sharp peak at 1332 cm^−1^ corresponds to the *sp*^3^-bonded carbon (“dia”) and a broadened peak at around 1360 cm^−1^ (D-band) corresponds to disordered carbon. The G-band corresponding to a nanographite phase is observed at around 1540 cm^−1^ [21]. It should be mentioned that the broadened diamond peak is normally observed for NCD films due to the small diamond grain size which is on the nanometer scale, and the presence of *sp*^2^ admixtures in the grain boundaries [22,23]. Moreover, the Raman spectrum shows an *I*_D_/*I*_G_ ratio of 1.13, related to the size of the graphite clusters in the NCD films [24], which is higher than that of the *I*_D_/*I*_G_ value of NCD/Cr/Si films (*I*_D_/*I*_G_ = 0.85, spectrum II, Figure S3 of Supplementary Materials). The increase of the *I*_D_/*I*_G_ value implies the formation of nanographite and a decrease in *sp*^3^ content according to a three stage model of increasing disorder in carbon materials [24,25] (i.e., there is a conversion of *sp*^3^ to *sp*^2^ content in NCD/Cr/LNO films as compared with the NCD/Cr/Si films).

The surface bonding characteristics of the NCD films were investigated using XPS. The C1s photoemission spectrum of NCD/Cr/LNO films (Figure S4c in Supplementary Materials) shows that these films contain the *sp*^3^ C–C peak of 35.4% with *sp*^2^ C=C peak intensity of 53.9% and CO/C–O–C peak intensity of 10.7%, respectively. In contrast, for the NCD/Cr/Si films, the *sp*^3^ C–C peak intensity is around to 47.0%, the *sp*^2^ C=C peak intensity is around to 45.4%, and the CO/C–O–C peak is around 7.6% (Figure S4b in Supplementary Materials). There is a larger *sp*^2^ content for NCD/Cr/LNO films as compared with that for NCD/Cr/Si films. Moreover, the C1s spectrum of NCD/Cr/LNO is shifted towards the low energy side relative to that of the NCD/Si films (Figure S4a in Supplementary Materials) [26], indicating the formation of more nanographitic phases in the NCD/Cr/LNO films.

**Figure 3:**
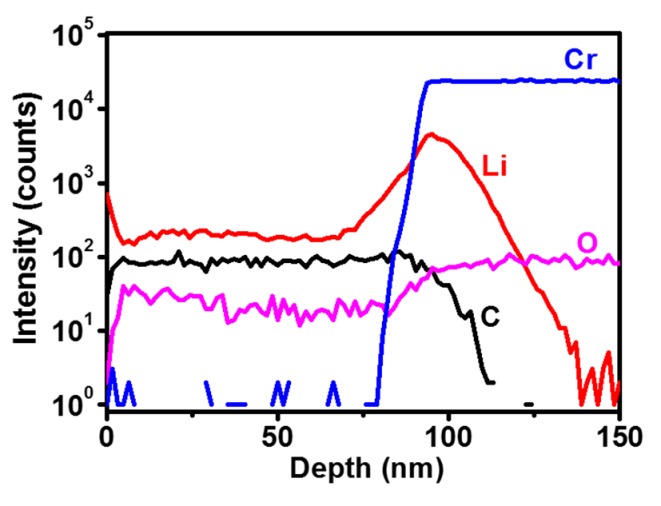
Secondary ion mass spectroscopy (SIMS) depth profiles of C, Li, Cr, and O species in NCD/Cr/LNO films.

It is evident through the Raman and XPS studies that there is a more abundant presence of nanographitic phases in the NCD/Cr/LNO films as compared to the NCD/Cr/Si films. The nanographitic phase is located along the grain boundaries of the NCD/Cr/LNO films, which provides conducting paths for the efficient transport of electrons. This is the key factor ensuing in the better conductivity and superior FEE properties for NCD/Cr/LNO films. The question that still remains is what are the roles of the Li incorporation and the presence of the Cr interlayer on the enhanced conductivity and FEE behavior of the NCD/Cr/LNO films? For a good field electron emitter, a sufficient electron supplies from the substrate to the emitting sites is very crucial, in addition to the low work function of the emitting surface. The conductivity of the diamond and the resistance of the diamond-to-substrate interface need to be optimized for an efficient supply of electrons. The results described above indicate that the utilization of a Cr interlayer and incorporation of Li in NCD films fulfill these two critical requirements simultaneously, such that NCD/Cr/LNO films show a low bulk/interfacial resistivity leading to enhanced FEE properties. To understand the genuine factor enhancing these properties, the elemental depth profile analysis of the NCD/Cr/LNO films was carried out using SIMS. The depth profile in Figure 3 proposes that Li is mostly present between diamond and the Cr interlayer, indicating that the Li from the LNO substrates can diffuse through the Cr interlayer and be incorporated into the NCD films to a depth of about 90 nm. The Li has thus been doped into the whole NCD film. O diffusion is also observed, but in a much lower level [27]. The presence of Cr at the interface region can lead to reactions with the carbon species in the growth plasma, forming chromium carbide [16,28,29,30], that can suppress the formation of amorphous carbon (*a*-*C*), inducing the formation of diamond nuclei, and at the same time provide an enhanced bonding to the LNO substrate. The Cr interlayer interacts very well with both the NCD and LNO materials, not only improving the adhesion but also suppressing the diffusion of oxygen into the NCD films, while allowing the Li to diffuse through and be efficiently incorporated into the NCD films. Smith et al. [31] observed that Li atoms doped in the diamond films diffused markedly into the grain boundaries. Therefore, it is reasonable to assume that Li atoms are not doped inside the diamond grains but tend to aggregate or couple with other impurities in the NCD films. Restated, the diffused Li into the NCD films tend to reside at the grain boundaries, which can activate the formation of nanographitic phases, as like the case reported by Sankaran et al. [32], and thereby, forming conduction nanochannels that provide grain boundary transport. While such an assumption for the role of Li incorporation into NCD films on enhancing the conductivity of the materials sounds quite reasonable, further microstructural investigation using transmission electron microscopy is required to directly elucidate the formation of nanographitic phases at the grain boundaries due to Li doping.

Consequently, the enhancement in the FEE properties of NCD/Cr/LNO is believed to be due to the use of a Cr interlayer and the Li-induced formation of nanographitic phases at the grain boundaries. The former increases the adhesion of the NCD films, while lowering the resistance for the transport of electrons across the interface region. The efficient Li incorporation increases markedly the bulk conductivity of the materials. Once the electrons can pass through the diamond-to-Cr/LNO interface, they can transport easily through the nanographitic phases induced along the grain boundaries of the films, to the emitting surface [33,34].

Furthermore, to show the great potential of the NCD/Cr/LNO films for the device applications, an improved performance of a microplasma device using the NCD/Cr/LNO films as cathode is demonstrated. The detailed procedure for the fabrication of microplasma devices and the testing methodology for evaluation of the robustness of the cathode materials can be found in the supplementary information and Figure S5. Inset III of Figure 1 depicts a series of photographs of plasma devices at different applied voltages, showing that the microplasma using the NCD/Cr/LNO films as cathode can be triggered by a voltage of 310 V. The intensity of the PI images increases monotonically with the applied voltage. This is better illustrated by the increase of the plasma current for NCD/Cr/LNO based microplasma devices reaching 615 μA at an applied voltage of 550 V (Figure S5a). To evaluate the stability of the PI performance, the plasma current was monitored over a period of 150 min with a constant applied voltage of 350 V. Figure S5b reveals that the plasma current of 133 μA is upheld for a period of 143 min, showing a high life-time stability for these microplasma devices. The better plasma performance using NCD/Cr/LNO films as cathode can be ascribed to the superior FEE properties of said films.

## 4. Conclusions

In summary, Li doping into NCD films grown at a low temperature of 350 °C, using a Cr as interlayer, was accomplished via diffusion of Li from LNO substrates. The NCD/Cr/LNO films showed a low resistivity of 0.01 Ω·cm and enhanced FEE properties, viz. a low turn-on field of 2.3 V/μm, a high FEE current density of 11.0 mA/cm^2^ at *E* = 4.9 V/μm, a large field enhancement factor of 1670, and good life-time stability of 445 min at *J* = 3.0 mA/cm^2^. Such an enhancement in the field emission behavior originates from the unique material combination: (i) the diffused Li atoms into the NCD films induced the formation of nanographitic phases along the grain boundaries, leading to better conductivity for these materials; and (ii) the Cr interlayer not only enriched the adherence of the NCD films on the LNO substrate but also efficiently improved the transport of electrons crossing the diamond-to-LNO interface. Both factors result in enhanced FEE properties of the NCD/Cr/LNO films. Additionally, the potential application of such films is demonstrated by the PI measurements, where a lowering of the threshold voltage to 310 V and increased life-time stability of 143 min at a plasma current of 133 μA, was observed. Consequently, the superior electrical conductivity and the enhanced FEE and PI characteristics render the low temperature deposited Li-doped NCD films a potential candidate for applications in flat panel displays and high brightness electron sources.

## Figures and Tables

**Table 1:** Comparison of the field electron emission properties of various Li-incorporated diamond based field emitters.

Materials	Resistivity (Ω·cm)	Turn-on Field (V/µm)	FEE Current Density (mA/cm^2^)	Life-Time (min)
Li ion implanted NCD [14]	9 × 10^–2^	10.6	25.5 @ 23.2 V/µm	1090
Freestanding Li doped UNCD [15]	1.2	4.2	0.3 @ 10.0 V/µm	---
NCD/Si [Present study]	7.1 × 10^4^	21.3	4.8 @ 35.7 V/µm	88
NCD/Cr/Si [Present study]	4.5 × 10^3^	11.8	6.4 @ 20.0 V/µm	215
NCD/Cr/LNO [Present study]	1 × 10^–2^	2.3	11.0 @ 4.9 V/µm	445

## Supplementary Materials

The following are available online at http://www.mdpi.com/2079-4991/8/9/653/s1, Figure S1: (a) Field electron emission properties (current density-applied field, *J*-*E*, curves) measured in high vacuum environment with the inset showing the corresponding Fowler–Nordheim (F–N) plots and (b) shows the life-time stability measurements (*J*-time curves) for I. NCD/Si and II. NCD/Cr/Si films, Figure S2: SEM micrographs of (a) NCD/Si and (b) NCD/Cr/Si thin films, Figure S3: The micro-Raman (*λ* = 488.0 nm) spectra of (I) NCD/Si and (II) NCD/Cr/Si thin films, Figure S4: The XPS spectra of (a) NCD/Si, (b) NCD/Cr/Si, and (c) NCD/Cr/LNO thin films, Figure S5: (a) The plasma current - applied voltage curve, indicating the increase of plasma current with the applied voltage. (b) The plasma life-time measurements: the plasma emission current versus time, of a microplasma device, which utilized ITO coated glass as anode and using NCD/Cr/LNO films as cathode material.
