# Supplementary material for: Low Temperature Synthesis of Lithium-Doped Nanocrystalline Diamond Films with Enhanced Field Electron Emission Properties
Source: Nanomaterials (Basel). 2018 Aug 24;8(9):653. doi: 10.3390/nano8090653 (PMC6164399; doi:10.3390/nano8090653)
Supplement: Supplementary file 1 [file nanomaterials-08-00653-s001.pdf]

nanomaterials

Supplementary information

Low Temperature Synthesis of Lithium-Doped Nanocrystalline Diamond Films with Enhanced Field Electron Emission Properties

Kamatchi Jothiramalingam Sankaran [1,2,†,*], Kalpataru Panda [3,†], Ping-Yen Hsieh [4], Paulius Pobedinskas [1,2], Jeong Young Park [3,5], Marlies K Van Bael [1,2], Nyan-Hwa Tai [4], I-Nan Lin [6] and Ken Haenen [1,2,*]

This supplementary information contains the comparison on the field electron emission (FEE) properties and other materials characteristics of the NCD films grown either directly on Si (NCD/Si) or on Cr-coated Si substrates (NCD/Cr/Si) to illustrate the effect of the Cr interlayer on the characteristics of NCD films. There are 5 measurements, which include 1. the FEE properties (the *J*-E curves) along with the Fowler-Nordheim plots and the life-time (τ) stability measurements, 2. the SEM micrographs, 3. the Raman spectra, 4. the XPS spectra, 5. the characteristics of the microplasma devices: plasma current density versus time or against applied voltage curves and a comparative table on the field electron emission properties of various Li incorporated diamond based field emitters.

The four-point probe measurements for the NCD/Si and NCD/Cr/Si films indicated that the resistivity values of NCD/Si and NCD/Cr/Si films are 7.1×10^4 and 4.5×10^3 Ω·cm, respectively. Figure S1 shows the FEE properties of these films. The E_0 value, which is the turn-on field for the FEE process for NCD/Si films is around 21.3 V/μm (curve I, Figure S1a) and decreased to 11.8 V/μm for NCD/Cr/Si films (curve II, Figure S1a), whereas the *J* value increased from 4.8 mA/cm^2 (at an applied field of 35.7 V/μm) for NCD/Si films to a value of 6.4 mA/cm^2 (at an applied field of 20.0 V/μm) for NCD/Cr/Si films.

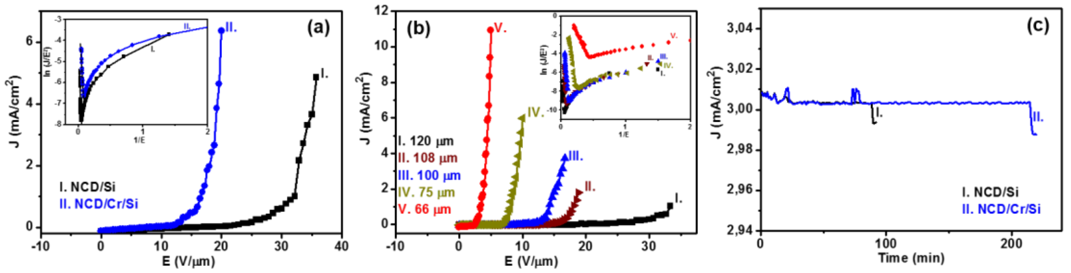

Figure S1. (**a**) Field electron emission properties (current density-applied field, *J*-E, curves) measured in high vacuum environment with the inset showing the corresponding Fowler-Nordheim (F-N) plots, (**b**) FEE characteristics measured on the NCD/Cr/LNO films for several d values with inset shows the corresponding F-N plots and (**c**) shows the life-time stability measurements (*J*-time curves) for I. NCD/Si and II. NCD/Cr/Si films.

Figure S1b shows the *J*-E curves with corresponding F-N plots measured for different cathode-anode separation "*d*" values for NCD/Cr/LNO films. We notice that, as expected, by increasing the distance of the tip from the surface, higher applied field are necessary to extract electrons from NCD/Cr/LNO films. The life-time (τ) stability measurements of NCD/Si and NCD/Cr/Si films were evaluated by measuring the *J* versus time curves. Figure S1c shows that the emission current density variations corresponding to *J* of 3.0 mA/cm^2 was recorded over a period of 215 min for NCD/Cr/Si films (at a working field of 18.9 V/μm), before the start of degradation (curve II, Figure S1c). In contrast, the NCD/Si films show emission current variations recorded for a

period of 88 min under the same test current density of 3.0 mA/cm^2 (at a working field of 34.0 V/μm) (curve I, Figure S1c), indicating that the presence of a Cr-interlayer not only enhances the FEE properties of the films, but also improves the lifetime stability of the materials.

Figure S2 shows the SEM micrographs of (a) NCD/Si and (b) NCD/Cr/Si thin films, which reveal that both films contain ultrasmall diamond grains with very smooth surface. The use of a Cr interlayer seems not markedly influence the morphology of the NCD films.

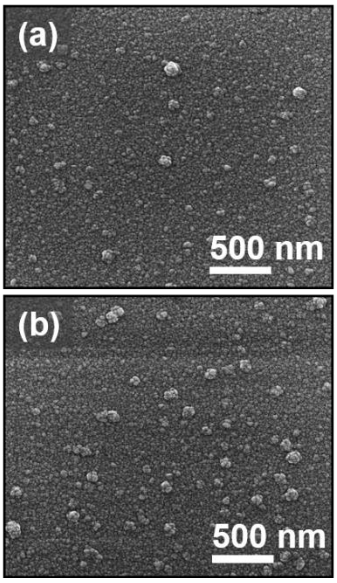

Figure S2. SEM micrographs of (**a**) NCD/Si and (**b**) NCD/Cr/Si thin films.

Figure S3 shows the micro-Raman spectra of (I) NCD/Si and (II) NCD/Cr/Si thin films. The two Raman spectra are essentially the same. Peaks at around 1190 cm^{-1} and 1470 cm^{-1} are ascribed to the ν_1 and ν_3-modes of *trans*-polyacetylene (*t*-PA) present at the grain boundaries. A sharp peak at 1334 cm^{-1} corresponds to sp^3-bonded carbon ("dia"), and a broadened peak at around 1360 cm^{-1} (D-band) corresponds to disordered carbon. The G-band is observed at around 1540 cm^{-1}. It should be mentioned that the broadened diamond peak is normally observed for NCD films due to the small diamond grain size and the presence of sp^2 admixtures in the grain boundaries. Moreover, the Raman spectra show a I_D/I_G ratio of 0.71 and 0.85 for NCD/Si films and NCD/Cr/Si films, respectively, implying the formation of nanographite and decrease in sp^3 content, i.e., there is conversion of sp^3 to sp^2 content in NCD/Cr/Si films compared with those in NCD/Si films.

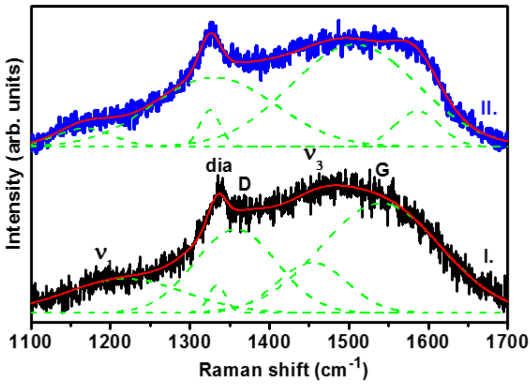

Figure S3. The micro-Raman (λ=488.0 nm) spectra of (I) NCD/Si and (II) NCD/Cr/Si thin films.

The surface bonding characteristics of the NCD films were investigated using X-ray photoelectron spectroscopy (XPS; PHI 1600). The measurements were conducted without ion sputtering etching to avoid reconfiguration of the bonds. The binding energies at 284.6, 285.2 and 286.6 eV are corresponding to the sp^2 C=C, sp^3 C–C and CO/C–O–C bonds, respectively. The C1s photoemission spectra of the XPS measurements for the NCD films are shown in Figure S4 to estimate the relative intensities of sp^3 and sp^2 components of NCD/Si, NCD/Cr/Si and NCD/Cr/LNO films. The binding energies at 284.6, 285.2 and 286.6 eV are corresponding to sp^2 C=C, sp^3 C–C and CO/C–O–C bonds, respectively. In NCD/Si films, sp^3 C–C bonding is predominant with a peak intensity of 67.5%, while sp^2 C=C intensity is 28.9% (Figure S4a) and the CO/C–O–C peak is seen with an intensity of 3.6%.

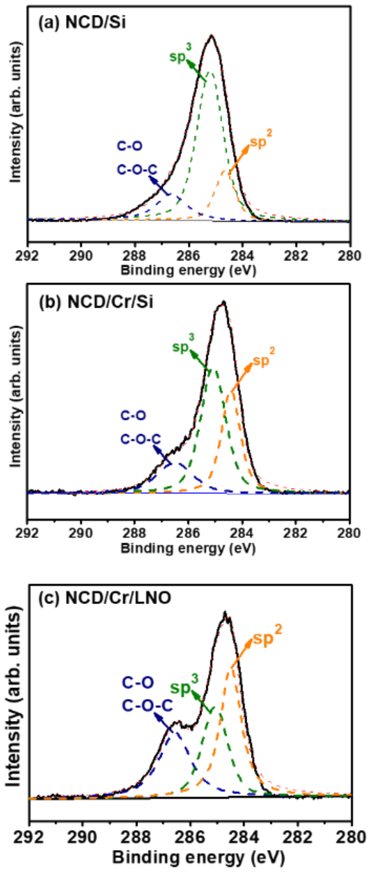

Figure S4. The XPS spectra of (**a**) NCD/Si, (**b**) NCD/Cr/Si and (**c**) NCD/Cr/LNO thin films.

For NCD/Cr/Si, sp^3 C–C peak intensity decreases to 47.0% and sp^2 C=C peak intensity increases to 45.4% (Figure S4b). There is a larger peak intensity of the CO/C–O–C for NCD/Cr/Si films (7.6%) compared with that for NCD/Si films (3.6%) that is due to the oxidation of Cr when exposed to air. The C1s photoemission spectrum of NCD/Cr/LNO films (Figure S4c) show that these films contain the sp^3 C–C peak of 35.4% with sp^2 C=C peak intensity of 53.9% and CO/C–O–C peak intensity of 10.7%. There is an even larger sp^2 content for NCD/Cr/LNO films as compared with that for NCD/Cr/Si films. Moreover, the C1s spectrum of NCD/Cr/LNO is shifted towards the low energy side compared to the NCD/Si, indicating the formation of more nanographitic phases in the NCD/Cr/LNO films.

In the microplasma devices, an ITO coated glass was used as the anode. The cathode-to-anode separation was fixed by a teflon spacer of 1.0 mm in thickness. A circular hole of about 3.0 mm in

diameter was cut out from the teflon spacer to form a microcavtiy. The device was placed in a stainless steel chamber, which was evacuated to a base pressure of 0.1 mTorr and was then purged with Ar for 10 min. Argon gas was channeled into the chamber at a flow rate of 30 sccm and the chamber pressure was maintained as 25 Torr throughout the measurements. A pulsed direct current voltage in bipolar pulse mode (20 ms square pulse, 6 kHz repetition rate) was used to trigger the plasma. Notably, the microplasma is the harshest environment for cathode emitters, as these materials are exposed to energetic Ar-ion bombardment in these devices.

The inset of Figure S5a depicts a series of photographs of the plasma devices at different applied voltages. These photographs show that the microplasma devices using the NCD/Cr/LNO films as cathode can be triggered by a voltage of 310 V and the intensity of the PI images increases monotonously with the applied voltage. This is better illustrated by the monotonous increase of the plasma current for NCD/Cr/LNO films based microplasma devices that reaches 615 μA at an applied voltage of 550 V (Figure S5a). To evaluate the stability of the PI performance for the NCD/Cr/LNO films based microplasma devices, the plasma current was monitored over a long period with a constant applied voltage of 350 V. The plasma current of 133 μA is upheld for a period of 143 min that shows a high life-time stability (Figure S5b).

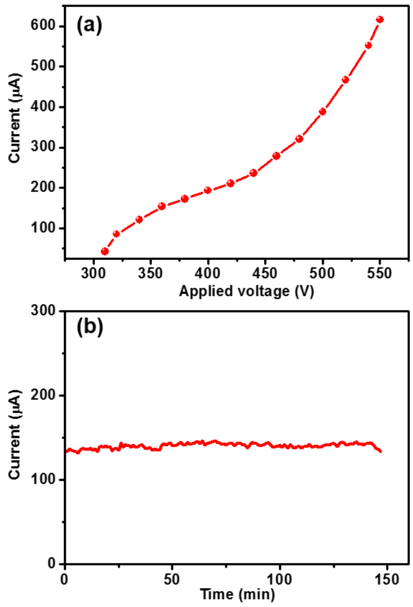

Figure S5. (**a**) The plasma current - applied voltage curve, indicating the increase of plasma current with the applied voltage. (**b**) The plasma life-time measurements: the plasma emission current versus time, of a microplasma device, which utilized ITO coated glass as anode and using NCD/Cr/LNO films as cathode material.

© 2018 by the authors. Submitted for possible open access publication under the terms and conditions of the Creative Commons Attribution (CC BY) license (http://creativecommons.org/licenses/by/4.0/).
